# Real world report: PSMA-PET/CT-guided TNM restaging and its consequences for treatment planning in prostate cancer

**DOI:** 10.3389/fonc.2026.1756827

**Published:** 2026-04-15

**Authors:** Han Chen, Peng Xian, Nan Liu, Yanping Song

**Affiliations:** Department of Urologic Oncology Surgery, Chongqing University Cancer Hospital (Chongqing Cancer Institute & Chongqing Cancer Hospital), Chongqing, China

**Keywords:** CTNM, MDT, prostate cancer, prostate-specific membrane antigen, treatment planning

## Abstract

**Objective:**

This study aimed to explore the effect of prostate-specific membrane antigen (PSMA) positron emission tomography and computed tomography (PET/CT) on clinical TNM (cTNM) staging assessment and treatment decision-making in prostate cancer,providing real-world evidence for its clinical application in China.

**Methods:**

We retrospectively analyzed the clinical data of 125 prostate cancer patients who underwent PSMA PET/CT at Chongqing University Cancer Hospital from January 2019 to June 2022. The results were compared with traditional imaging methods (bone scan, CT, MRI) in the overlapping subset of patients with both examinations to assess changes in TNM staging and treatment plans.

**Results:**

After PSMA PET/CT, TNM staging changed in 48 patients (38.40%), particularly in the primary prostate cancer and pre-radical surgery groups regarding regional lymph node detection (P = 0.007, P = 0.040). CTNM staging was revised in 36 patients (28.80%), most significantly in the post-radical surgery and mCRPC groups (47.06% and 55.88%, respectively; P = 0.036, P = 0.000). Changes in treatment plans did not reach statistical significance for newly diagnosis of Prostate and pre-radical surgery group (P = 0.222,P= 0.151).

**Conclusion:**

PSMA PET/CT significantly impacts cTNM staging assessment and treatment decisions in prostate cancer, offering precise evaluation and auxiliary diagnosis.

## Introduction

Prostate cancer is the second most common malignancy among men globally (1). While its incidence in China is lower than in Western countries, it has been rising rapidly in recent years (2), with a notably lower prognosis compared to Western nations (3), due to a high proportion of middle-advanced stages at diagnosis, insufficient early screening, and non-standard conventional imaging staging in primary medical institutions. Accurate staging and timely treatment adjustments are crucial for improving patient outcomes. Traditional imaging methods have limited diagnostic efficacy, necessitating more advanced techniques (4).

PSMA, highly expressed in prostate adenocarcinoma, is a key target for diagnosis and treatment (5). PSMA PET/CT, using 68-gallium as a marker, has shown significant diagnostic efficacy across various stages of prostate cancer (6), enhancing the level of diagnosis and treatment. Although numerous international studies (including randomized prospective evidence) have verified the diagnostic value of PSMA PET/CT, real-world studies targeting Chinese population characteristics are still scarce, and the actual impact of PSMA PET/CT on cTNM staging revision and treatment decision-making in Chinese clinical practice remains to be further clarified. This study retrospectively analyzed the clinical data of 125 prostate cancer patients who underwent PSMA PET/CT to evaluate its impact on cTNM staging revision and treatment decisions. The study aims to fill the gap of localized real-world data for PSMA PET/CT application in China and provide actionable evidence for its tiered clinical promotion in Chinese medical institutions at all levels.

## Methodology

### Clinical information

#### Eligibility and exclusion criteria

Inclusion criteria: ① Pathologically confirmed prostate cancer patients treated in Chongqing University Cancer Hospital from January 2019 to June 2022; ② Patients who completed PSMA PET/CT examination and at least one conventional imaging examination (bone scan, CT, MRI, transrectal ultrasonography); ③ Complete clinical, imaging, and follow-up data available in the electronic medical record system; ④ Patients who provided informed consent for clinical data collection and research use (in line with the ethical approval of the hospital).Exclusion criteria: ① Patients with other primary malignant tumors; ② Patients with severe organ dysfunction (heart, liver, kidney) that cannot tolerate imaging examinations; ③ Patients with incomplete imaging or clinical data that affect staging and treatment plan evaluation; ④ Patients with PSMA PET/CT image quality that does not meet diagnostic requirements due to technical reasons or patient factors (e.g., severe motion artifact).

#### Treatment plan data collection and analysis

All treatment plan information was uniformly retrieved from the hospital’s standardized electronic medical record system (EMRS) and the MDT consultation archive system. The MDT consultation records (including urology, oncology, nuclear medicine, radiology, and radiotherapy) before and after PSMA PET/CT examination were the gold standard for collecting treatment plan information, which included written diagnosis and treatment suggestions, treatment decision-making meeting minutes, and doctor-patient communication records. Two independent urologic oncology physicians cross-checked the initial and revised treatment plans; discrepancies were resolved by a third senior physician. Treatment modification was defined as any change in the type, scope, or sequence of treatment (e.g., adding local radiotherapy/surgery, changing systemic therapy regimens, adjusting the order of comprehensive treatment). The treatment modification rate was calculated as the number of patients with revised treatment plans divided by the total number of included patients, and subgroup analysis was performed according to the disease progression grouping.

#### The specific timing of ^68^Ga-PSMA PET/CT examination at each disease stage of the included patients

The clinical management workflow of prostate cancer patients included in this study strictly followed the 2019–2022 Chinese Clinical Guidelines for Prostate Cancer and the standardized diagnosis and treatment protocol of Chongqing University Cancer Hospital (a tertiary cancer center in China). The overall diagnostic-therapeutic pathway was: pathological confirmation of prostate cancer → initial clinical evaluation (serum PSA, physical examination, conventional imaging) → initial cTNM staging and preliminary treatment plan formulation via MDT consultation → ^68^Ga-PSMA PET/CT examination → re-evaluation of staging via MDT combined with PET/CT results → revision of treatment plan (if necessary) → implementation of treatment and follow-up.

#### Image analysis and diagnostic criteria section

The image analysis of ^68^Ga-PSMA PET/CT was jointly completed by two experienced nuclear medicine physicians and two radiologists (with more than 10 years of experience in prostate cancer imaging diagnosis) in a double-blind manner, and the analysis combined visual assessment as the main method and semi-quantitative analysis (SUVmax) as an auxiliary diagnostic index.

We collected data from 125 prostate cancer patients (aged 51–91 years, mean 71.02 ± 8.60 years, Shapiro-Wilk test P = 0.236) confirmed pathologically at Chongqing University Cancer Hospital from January 2019 to June 2022. Their serum PSA levels ranged from 0.010 to 2,389.290 ng/mL (median 14.54 ng/mL, P25-P75: 4.32-56.89 ng/mL, Shapiro-Wilk test P<0.001). Patients were divided into four groups based on disease progression and PSMA PET/CT timing: newly diagnosed (38), pre-radical surgery (36), post-radical surgery (17), and mCRPC (34). We compared PSMA PET/CT findings with traditional imaging (bone scan, CT, MRI, ultrasound) to assess TNM staging changes and treatment plan modifications.

### Conventional imaging

The selection of conventional imaging examinations was strictly based on the 2019–2022 Chinese Clinical Guidelines for Prostate Cancer and individual patient conditions (e.g., serum PSA level, clinical symptoms, initial staging). Bone scans were mainly used for patients with PSA >10 ng/mL or suspected bone metastasis, and chest CT was prioritized for patients with respiratory symptoms or suspected visceral metastasis, which is consistent with clinical real-world practice in China’s tertiary cancer centers and avoids over-examination.

Among the 125 patients, 45 underwent 99Tcm-phosphate bone scanning, 42 had chest/abdominal CT, and 48 underwent transrectal ultrasonography of the prostate. All patients underwent pelvic mpMRI using a 3.0T superconducting MRI system, including high-resolution T2-weighted imaging, diffusion-weighted imaging, and T1-weighted dynamic enhancement scans.

### PSMA PET-CT examination

The 68Ga-PSMA-11 PET/CT scans were performed using a GE Discovery 710 PET/CT scanner. Patients received an intravenous injection of 2 MBq/kg of the tracer, followed by imaging 60 minutes post-injection. The scanning range covered from the head to the mid-femur, with PET acquisition time of 2 minutes per bed position. CT data were used for attenuation correction, and images were reconstructed using an iterative algorithm to generate multi-planar PET/CT fusion images.

### Image analysis and diagnostic criteria

Two experienced nuclear medicine and imaging physicians jointly reviewed the images. Pathological results were the gold standard for lesions undergoing surgical resection or biopsy. For other lesions, clinical findings, serum PSA levels, or imaging follow-up changes were used as diagnostic criteria. Malignant lesions were determined based on typical imaging features, significant progression during follow-up, or significant reduction after treatment.

### Clinical staging and treatment planning

TNM staging followed the 8th edition of the American Joint Committee on Cancer TNM classification. Multidisciplinary consultations formulated TNM staging and treatment plans before and after PSMA PET/CT. Data were retrieved via the electronic medical record system.Nx and Mx indicated that regional lymph node or distant metastasis was indeterminate/inconclusive on conventional imaging, due to non-specific findings, equivocal lesions, or limited spatial resolution of routine imaging modalities. The initial cTNM staging was determined based on conventional imaging results, and PSMA PET/CT revised the cTNM staging by detecting lymph node or distant metastases missed by conventional imaging, without altering the AJCC 8th edition TNM staging criteria itself.

### Statistical analysis

SPSS 25.0 was used for statistical analysis. Normally distributed data were described as mean ± SD, non-normally distributed data as median (P25, P75), and categorical data as frequencies or percentages. Comparisons of TNM staging and treatment plan changes used chi-square or Fisher’s exact tests, with P < 0.05 indicating statistical significance.

## Results

### Lesion detection rate comparison

All comparisons were performed in the overlapping subset of patients with both PSMA-PET/CT and corresponding conventional imaging. PSMA PET/CT showed significantly higher detection rates than conventional imaging in the matched patient subsets for primary lesions (97.14% vs. 93.33%), pelvic lymph node metastases (43.20% vs. 24.00%), extraregional lymph node metastases (4.80% vs. 1.60%), bone metastases (57.60% vs. 45.60%), and visceral metastases (8.80% vs. 4.80%). The difference in regional lymph node detection was statistically significant (P = 0.001).The lack of statistical significance in the detection rate of other lesion types was due to the small sample size of patients with such metastases (e.g., only 6 patients with extraregional lymph node metastasis and 11 patients with visceral metastasis in the study population) (See **Table 1**).

**Table 1:** Comparison of TNM staging before and after PET/CT of 125 cases of PSMA, total 48 patients with changed staging.

Group and number of cases		T staging				P	N staging			p	M staging					P
		TX	T2	T3	T4		Nx	N0	N1		Mx	M0	M1a	M1b	M1c	
Group I: Post-radical surgery group Number of cases (n=17)	Conventional imaging	1	7	8	1	P=1.00	0	15	2	P=0.398	1	12	0	4	0	P=0.486
	PSMA	1	7	8	1		0	12	5		0	11	0	6	0	
Group II: First Diagnosis of Prostate Cancer Group Number of cases (n=38)	Conventional imaging	0	13	11	14	P=0.961	2	24	12	P=0.007	2	12	0	21	3	P=0.214
	PSMA	0	13	12	13		0	13	25		0	7	2	25	4	
Group III: Pre-radical surgery group Number of cases (n=36)	Conventional imaging	3	17	15	1	P=0.897	0	36	0	P=0.040	0	27	0	9	0	P=0.230
	PSMA	2	18	14	2		0	32	4		2	22	0	12	0	
Group IV: mCRPC group Number of cases (n=34)	Conventional imaging	11	2	3	18	P=1.000	3	15	16	P=0.176	1	11	2	17	3	P=0.236
	PSMA	11	2	3	18		0	14	20		0	5	4	18	7	
Total Number of cases (n=125)	Conventional imaging	15	39	37	34	P=0.997	5	90	30	P=0.001	4	62	2	51	6	P=0.102
	PSMA	14	40	37	34		0	71	54		2	45	6	61	11	

Bold values indicate statistically significant findings (generally P < 0.05).

### CTNM staging comparison

All cTNM staging revisions were based on the AJCC 8th edition criteria, with the revision reason being the detection of missed metastases by PSMA-PET/CT.After PSMA PET/CT, 48 patients (38.40%) experienced cTNM staging revisions. Specifically, T staging changed in 3 patients (2.40%, 2 upstaged, 1 downstaged), N staging in 24 patients (19.20%, all upstaged), and M staging in 21 patients (16.80%, 19 upstaged, 2 downstaged). Five patients (4.00%) had changes in two staging categories. The N staging changes were statistically significant compared to conventional imaging (P < 0.05), particularly in the newly diagnosed and pre-radical surgery groups (P = 0.007, P = 0.040).meanwhile, 4 patients with N0 by conventional imaging were confirmed as N1 in pre-radical surgery group (See **Table 1**).

### Treatment plan comparison

Treatment plans changed in 36 patients (28.80%) after PSMA PET/CT. Specifically, 18 patients (14.40%) added local treatment (surgery or radiotherapy), 11 patients (8.80%) changed systemic treatment and added local treatment, and 7 patients (5.60%) changed systemic treatment alone. The highest change rates were in the post-radical surgery and mCRPC groups (47.06% and 55.88%, respectively; P = 0.036, P = 0.000). The newly diagnosed group had the lowest change rate (5.26%). Changes in treatment plans did not reach statistical significance for newly diagnosed prostate cancer and pre-radical surgery group(P = 0.222,P= 0.151) (See **Table 2**).

**Table 2:** The treatment regimens of 36 patients were changed after 125 PSMA PET/CT examinations.

Group and number of cases		Treatment plan								P-value
		Follow up	Systemic therapy	Alteration of systemic therapy	Alteration of systemic therapy + localized therapy	Localized treatment	Radical prostatectomy	Radical surgery + pelvic lymph node dissection	Radical surgery + pelvic lymph node dissection + local radiotherapy	
Group I:Post-radical surgery group Number of cases (n=17)	conventionall Imaging	9	5	0	2	1	0	0	0	P=0.036
	PSMA	4	2	0	10	1	0	0	0	
Group II: First Diagnosis of Prostate Cancer Group Number of cases (n=38)	conventional Imaging	0	37	0	0	0	1	0	0	P=0.222
	PSMA	0	36	0	0	2	0	0	0	
Group III: Pre-radical surgery group Number of cases (n=36)	Conventional Imaging	0	0	0	0	0	8	23	5	P=0.151
	PSMA	0	0	0	0	0	6	18	12	
Group IV: mCRPC group Number of cases (n=34)	conventional Imaging	0	34	0	0	0	0	0	0	P=0.000
	PSMA	0	15	7	6	6	0	0	0	

Bold values indicate statistically significant findings (generally P < 0.05).

## Discussion

Prostate cancer in China has distinct clinical characteristics compared with Western countries, including late diagnosis, non-standard conventional staging in primary hospitals, and low popularization of advanced imaging techniques such as PSMA-PET/CT. These characteristics make the real-world research on PSMA-PET/CT in Chinese patients particularly important for clinical practice. The rapid increase in prostate cancer incidence in China, coupled with lower prognosis compared to Western countries, underscores the need for accurate staging and timely treatment adjustments. Traditional imaging methods (bone scan, CT, mpMRI) have limitations in detecting micrometastases, while PSMA PET/CT offers superior diagnostic efficacy, recognized worldwide. It was FDA-approved in December 2020 and recommended by European guidelines for postoperative biochemical recurrence (7).

### Sensitivity analysis on conventional imaging utilization rate

The relatively low utilization rate of chest CT and bone scans in the study population is a characteristic of real-world clinical practice rather than a research design defect. This setting is consistent with the research objective of exploring the clinical application value of PSMA PET/CT in Chinese real-world prostate cancer management. The subgroup analysis of 32 patients with complete conventional imaging examinations has further excluded the bias caused by incomplete conventional imaging, confirming the robustness of the study conclusions.

Our study demonstrated a statistically significant higher detection rate of regional lymph node metastases and a numerically higher detection rate of distant metastases with PSMA PET/CT than conventional imaging, leading to TNM staging changes in 38.40% of patients, mainly due to N and M upstaging. For the N stage, this was particularly significant in newly diagnosed and pre-radical surgery groups (P = 0.007, P = 0.040). Similar results (8) were reported by Dekalo et al., showing 82% accuracy for PSMA PET/CT in diagnosing regional lymph node metastasis. Domestic studies by Liu Lei et al (9). and Li Yajian et al (10). also confirmed the superior sensitivity and specificity of PSMA PET/CT compared to mpMRI in detecting pelvic lymph node metastasis. For the T stage and M stage, Sonia et al (11) found that the detection rate of primary lesion of PSMA PET/CT, mpMRI and PSMA PET/CT + mpMRI were 85%, 83% and 87%.And the diagnostic efficacy of PSMA PET/CT for primary lesions, metastatic lesions, and the assessment of whether the periprostatic tissue is invaded is higher than that of mpMRI (12), and the diagnostic efficacy of PSMA PET/CT for bone metastases is also higher than that of (99)Tcm-phosphate bone scan (13), our study showed that PSMA PET/CT had less impact on primary lesion (T stage) changes. This might be because the primary lesions of the post - radical surgery group and part of the mCRPC group had already been resected. The above studies and the results of the present study suggest that compared with conventional imaging, PSMA PET/CT is more sensitive to the higher TNM staging of prostate cancer, especially in the newly diagnosed prostate cancer group and in the pre-radical surgery group.

Treatment plans changed in 28.80% of patients after PSMA PET/CT, consistent with Davies et al.’s findings of 43% treatment changes (14). The treatment plan revision rate of mCRPC group in this study (55.88%) is higher than that of international studies, which is due to the high proportion of multi-line treatment-refractory patients in Chinese mCRPC population, and PSMA-PET/CT can detect more missed metastases to guide treatment adjustment. The highest changes were in post-radical surgery and mCRPC groups (47.06% and 55.88%, respectively), aligning with Koerber et al.’s (15) results of over 50% treatment changes in high-risk prostate cancer patients. The changes primarily involved adding local treatment or altering systemic therapy. These changes provided patients with more treatment options. However, it is still unknown if there is positive influence of these changes on progression-free survival, overall survival, and quality of life, which requires further prospective, large-sample study to validate.

PSMA-PET/CT is not covered by China’s national medical insurance, with a single scan costing 5,000–8,000 RMB and only available in tertiary hospitals. Stratified cost-effectiveness analysis shows it is cost-effective for metastatic castration-resistant prostate cancer (mCRPC) and post-radical surgery patients, as it can accurately localize metastases, avoid ineffective treatment and reduce overall costs. For low-risk newly diagnosed patients, conventional imaging is sufficient and more economical. A stratified application strategy is proposed: tertiary hospitals use PSMA-PET/CT for high-risk patients, while primary hospitals adopt conventional imaging and refer patients to tertiary hospitals when necessary.

Despite its excellent diagnostic performance, PSMA-PET/CT has limitations in prostate cancer detection: it has low detection rates for poorly differentiated or PSMA-low-expressing tumors, may produce false positives in benign lesions such as prostatitis and benign prostatic hyperplasia, has limited ability to detect micrometastases smaller than 5 mm, and has inter-observer variability in image interpretation affected by clinical experience.

In the PSMA-PET/CT era, conventional imaging remains indispensable in China: pelvic multiparametric MRI (mpMRI) is the gold standard for primary tumor T staging, 99Tcm-phosphate bone scan (about 1,000 RMB per scan) is the first-line screening for bone metastasis in primary hospitals, and chest/abdominal CT verifies visceral metastases, with their combination being the optimal staging strategy. This single-center retrospective study has limitations including limited sample size, incomplete baseline data and lack of long-term follow-up; future research should focus on multi-center prospective studies, long-term survival follow-up, health economic analyses for insurance inclusion, and machine learning models to improve image interpretation accuracy.

## Conclusion

PSMA PET/CT has a statistically significant higher detection rate of regional lymph node metastasis in prostate cancer than conventional imaging, and a numerically higher detection rate for primary lesions, distant lymph node metastases, bone metastases and visceral metastases. It is a valuable tool for precise evaluation and auxiliary diagnosis of prostate cancer. It provides detailed information on tumor invasion and metastasis, guiding treatment decisions across all stages of the disease. However, further studies are needed to assess its impact on patient outcomes in real world.

## Data Availability

The original contributions presented in the study are included in the article/supplementary material. Further inquiries can be directed to the corresponding authors.
